# Time to clearance of abdominal septic focus and mortality in patients with sepsis

**DOI:** 10.5935/0103-507X.20200029

**Published:** 2020

**Authors:** Rafael Barberena Moraes, Thiago Ferreira Serafini, Josi Vidart, Miriane Melo Silveira Moretti, Jaqueline Sangiogo Haas, Alan Pagnoncelli, Marco Aurélio Abreu Azeredo, Gilberto Friedman

**Affiliations:** 1 Hospital de Clínicas de Porto Alegre, Universidade Federal do Rio Grande do Sul - Porto Alegre (RS), Brasil.; 2 Programa Intrahospitalar de Combate à Sepse, Hospital de Clínicas de Porto Alegre, Universidade Federal do Rio Grande do Sul - Porto Alegre (RS), Brasil.; 3 Programa de Pós-Graduação em Ciências Pneumológicas, Universidade Federal do Rio Grande do Sul - Porto Alegre (RS), Brasil.; 4 Universidade Federal do Rio Grande do Sul - Porto Alegre (RS), Brasil.

**Keywords:** Sepsis, Septic shock, Hospital mortality, Intraabdominal infections/complications, Infections, Sepse, Choque séptico, Mortalidade hospitalar, Infecções intra-abdominais/complicações, Infecções

## Abstract

**Objective:**

To assess the relationship between time to focus clearance and hospital mortality in patients with sepsis and septic shock.

**Methods:**

This was an observational, single-center study with a retrospective analysis of the time to clearance of abdominal septic focus. Patients were classified according to the time to focus clearance into an early (≤ 12 hours) or delayed (> 12 hours) group.

**Results:**

A total of 135 patients were evaluated. There was no association between time to focus clearance and hospital mortality (≤ 12 hours versus > 12 hours): 52.3% versus 52.9%, with p = 0.137.

**Conclusion:**

There was no difference in hospital mortality among patients with sepsis or septic shock who had an infectious focus evacuated before or after 12 hours after the diagnosis of sepsis.

## Abstract (pt)

**Objetivo:**

Aferir a relação entre tempo para evacuação de foco e mortalidade hospitalar em portadores de sepse e choque séptico.

**Métodos:**

Estudo observacional, unicêntrico, com análise retrospectiva do tempo para evacuação de foco séptico abdominal. Os pacientes foram classificados conforme o tempo para evacuação do foco em grupo precoce (≤ 12 horas) ou tardio (> 12 horas).

**Resultados:**

Foram avaliados 135 pacientes. Não houve associação entre tempo para evacuação do foco e mortalidade hospitalar (≤ 12 horas versus > 12 horas): 52,3% versus 52,9%, com p = 0,137.

**Conclusão:**

Não houve diferença na mortalidade hospitalar entre pacientes com sepse ou choque séptico que tiveram foco infeccioso evacuado antes ou após 12 horas do diagnóstico de sepse.

## INTRODUCTION

Sepsis is defined as a serious, life-threatening organ dysfunction caused by a dysregulated host response to infection. Sepsis is the leading cause of death in noncardiac intensive care units (ICU). In low-income countries, sepsis-related death rates are increased.^(1-6)^ The mortality of sepsis and septic shock in Brazilian ICU is worryingly high; it is higher than that in developed countries (55% *versus* 30%).^(7-9)^

The mortality of sepsis seems to decrease when antibiotics are started early, and every hour of delay in the use of antibiotics increases mortality. The Surviving Sepsis Campaign (SSC)^(10)^ recommends that all patients be evaluated early for sites of infection amenable to source control (focus clearance). However, the association between time to focus clearance and outcome in patients with sepsis is less studied^(11-13)^ and consequently receives less attention in the literature. In the SSC,^(10)^ for example, there are nine pages devoted to the discussion of antimicrobial therapy and only one page addressing source control. In developing countries, data on focus clearance and sepsis mortality are even more scarce. Source control includes all physical measures taken to eliminate sources of infection, control contamination and restore anatomy and function. It includes drainage of infected fluids, debridement of infected soft tissues, removal of infected devices or foreign bodies and correction of the anatomical derangement resulting from microbial contamination.

Despite progress in the recommendations for treating patients with sepsis, such as optimization of hemodynamic management and indications for early antibiotic use, the understanding of the impact of focus clearance in septic patients is incomplete.

The objective of the present study was to analyze the correlation between the time to focus clearance in patients with intra-abdominal sepsis and hospital deaths in a high-complexity ICU. We hypothesized that delays in abdominal septic focus clearance beyond 12 hours after the diagnosis of severe sepsis or septic shock are associated with higher hospital mortality.

## METHODS

This study was approved by the Research Ethics Committee of *Hospital de Clínicas de Porto Alegre* (HCPA) under number 2016-0317. Because this was an observational study, it was not necessary to obtain an Informed Consent Form.

Since 2013, the HCPA, where the study was conducted, has adhered to the tenets of the “Brazil against Sepsis” project, and it has created the Intrahospital Program for Combatting Sepsis (Programa Intrahospitalar de Combate à Sepse - PICS). Since that time, as part of the project, the HCPA has monitored the care of patients with sepsis and septic shock admitted to the ICU and has prospectively collected care data using a questionnaire standardized by the Latin American Sepsis Institute (Instituto Latino Americano de Sepsis - ILAS). The PICS consists of five physicians (three intensivists, one emergency physician and one physician from the rapid response team), three nurses (two working in the ICU and one working in the emergency department) and one scholarship recipient. The program collects patient data and has a managerial function, i.e., it generates protocols for sepsis care and suggests policies and care practices to be adopted by hospital management. The program does not directly participate in patient care, which instead is performed by the rapid response team, emergency physicians, intensivists and other professionals of the institution.

The HCPA is a public, tertiary university hospital. Approximately 95% of the care provided is funded by Brazil’s public health system (Sistema Único de Saúde - SUS), and the hospital is a regional reference center for high-complexity visits. The ICU consists of 45 beds, and the hospital has approximately 600 beds for adults.

The present study retrospectively analyzed the time to surgical intervention by reviewing the charts of patients with abdominal sepsis who were admitted to the ICU from May 16, 2013, to March 20, 2018. All data except the time to focus clearance were prospectively collected using the PICS. Patients with suspected infection focus, at least two criteria for systemic inflammatory response syndrome and at least one organ dysfunction (called severe sepsis by prior consensus) were considered to have sepsis. The following presentations were considered indicative of organ dysfunction: hypotension, if systolic blood pressure (SBP) is < 90mmHg or mean arterial pressure (MAP) is < 60mmHg; altered consciousness level; lactate > 2mmol/L; diuresis < 0.5mL/kg in 6 hours; partial pressure of oxygen/fraction of inspired oxygen ratio (PO_2_/FiO_2_) < 300 or thrombocytopenia < 100,000/uL. Patients who needed vasopressors despite adequate fluid resuscitation were considered to have septic shock.

Septic focus was considered abdominal according to the clinical suspicion described in the medical records; in this study, cases in which the intraoperative findings confirmed the presence of abdominal focus of infection (visceral secretion, inflammation or perforation, and collection with antimicrobial growth) were analyzed according to the indications of the clinical and imaging evaluations. The results of cultures collected during surgery were not verified.

The medical records of patients with sepsis or septic shock of abdominal origin who were admitted to the ICU (before or after clearance of the focus of infection) who had indications for and underwent some type of intervention for septic focus clearance (surgical drainage or image-guided drainage) were reviewed according to this diagnostic hypothesis. As suggested in the SSC 2012 guidelines,^(14)^ patients were included in the early focus clearance group when clearance occurred within 12 hours after the diagnosis of sepsis and were included in the delayed group when clearance occurred 12 hours after diagnosis. Patients who were diagnosed with sepsis during the septic focus clearance procedure (i.e., the procedure was not indicated by the diagnostic hypothesis of sepsis) and those for whom it was not possible to identify the time between the sepsis diagnosis and septic focus clearance were excluded. The temporal relationship between the diagnosis of sepsis and surgery was calculated by determining the time of sepsis diagnosis and the time of intervention for septic focus clearance. The time of focus clearance was considered the recorded time of the patient’s arrival at the operating room or the time the punctures were recorded. Other data collected for analysis were the date and time of sepsis, number of surgical interventions performed, affected region, sex, number of organ dysfunctions, Sequential Organ Failure Assessment (SOFA) score, arterial lactate level at the time of diagnosis and death. Hospital death was considered a primary outcome. Organ dysfunction was defined according to the SOFA scoring system, where dysfunction was considered when any of the evaluated systems scored at least 1 point on the SOFA or presented an increase in this score. The time to infusion of the first antibiotic dose was considered the time interval between the first antibiotic administration and the diagnosis of sepsis or septic shock. In many patients, the use of antibiotics preceded the diagnosis of sepsis or septic shock; thus, negative values were recorded in these cases.

A descriptive analysis was performed. Continuous variables are described as the mean and standard deviation or median and interquartile range, and categorical variables are described as absolute and relative frequencies. To identify differences between groups, chi-square or Fisher’s exact tests were performed for categorical variables, and the Mann-Whitney test was performed for continuous variables. The relationship between mortality and time to surgical intervention was analyzed by univariate and multivariate analysis. In the logistic regression, mortality was corrected for SOFA score, time of initiation of antibiotic therapy, need for mechanical ventilation and prevalence of septic shock because these variables are commonly associated with mortality. The variable organ dysfunction was not included in the multivariate regression model because it showed high collinearity with SOFA score. The level of significance was set at 0.05. A post hoc analysis of lethality was performed at intervals of 12 hours for up to 36 hours.

The program Epi Info™ version 7 was used to calculate the initial sample size. The relative risk for the selected outcome (hospital mortality) in relation to focus clearance is 3.7 (27% for ≥ 6 hours and 9% for < 6 hours).^(11)^ Thus, the calculated sample size was 164, considering a power of 80% and a significance level of 5%.

Statistical analysis was performed using the Statistical Package for Social Science (SPSS). No data imputation method was used for missing data.

## RESULTS

Of the sample of 2,020 patients, 416 (20%) were diagnosed with abdominal sepsis. Procedures for focus clearance were identified in 142 patients, and the temporal relationship between the diagnosis of sepsis and focus clearance was determined for 135. Table 1 shows the general characteristics of the patients.

**Table 1:** Cohort profile according to the time to focus clearance (≤ 12 hours versus > 12 hours)

	Early (≤ 12 hours) n = 65	Delayed (> 12 hours) n = 70	p value
Age (years)	60.95 ± 13.42	61.17 ± 13.68	0.92
Male sex	34 (52.3)	39 (55.7)	0.73
Organ dysfunctions	3.35 ± 1.38	2.74 ± 1.28	0.01
SOFA score	8 [7 - 11]	6 [3 - 10] *n = 67	0.01
Lactate	3.4 [1.9 - 4.8] *n = 63	2.6 [1.59 - 5.2] *n = 58	0.59
Time to start of antibiotic therapy	0.5 [ -1.6 - 1.7] *n = 64	0.6 [ -3.1 - 2.4] *n = 66	0.73
Abdominal region affected			
Intestine	51 (60)	40 (57.1)	0.25
Liver/biliary tract	4 (6.2)	12 (17.1)	
Urinary tract	2 (3.1)	0	
Other	8 (12.3)	18 (25.7)	
Number of interventions performed			
1	39 (60)	42 (60)	0.23
2	10 (15.45)	16 (22.9)	
3	7 (10.8)	7 (10)	
≥ 4	9 (13.8)	5 (7.1)	
Focus clearance time (hours)	6 [4 - 9]	41.6 [19.9 - 107]	0.01
Need for mechanical ventilation	61 (93.8)	46 (65.7)	0.005
Septic shock	53 (81.5)	38 (54.3)	0.001
Hospital mortality	34 (52.3)	37 (52.9)	0.13

SOFA - Sequential Organ Failure Assessment Score.

* Indicates missing data. The results are expressed as the mean ± standard deviation, n (%) or median [interquartile range].

**Figure 1:**
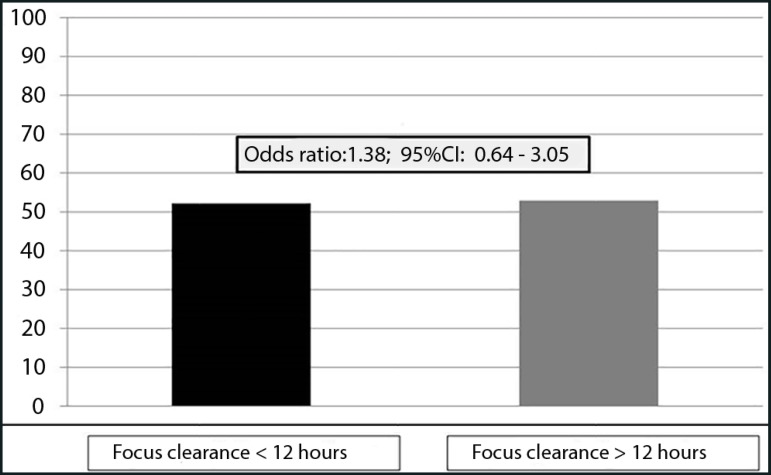
Mortality (%) according to time to septic focus clearance (≤ 12 hours versus > 12 hours). 95%CI - 95% confidence interval.

There was no difference in mortality between patients with early or delayed surgical intervention (52.3% *versus* 52.9%; p = 0.137) (Figure 1) in either the univariate analysis (odds ratio (OR) = 0.98; 95% confidence interval (95%CI) = 0.49 to 1.9) or in the multivariate analysis (OR = 1.38; 95%CI 0.64 to 3.05). Patients who underwent focus clearance within less than 12 hours had a higher SOFA score, more organ dysfunctions, an increased need for mechanical ventilation and a higher prevalence of septic shock (Table 2).

**Table 2:** Variables included in the multivariate analysis

	Odds ratio	95%CI
Time to focus clearance (≤ 12 hours versus > 12 hours)	1.38	0.64 - 3.05
Mechanical ventilation	3.17	1.08 - 10.22
SOFA score	0.98	0.87 - 1.1
Time to start of antibiotic therapy	1	0.98 - 1.03
Septic shock	1	0.41 - 2.43

95%CI - 95% confidence interval; SOFA - Sequential Organ Failure Assessment.

Even after the patients were stratified into more groups according to the elapsed time from diagnosis to intervention, there was only a higher proportion of deaths in the group for which the time was longer than 36 hours.

## DISCUSSION

In this observational study, focus clearance within 12 hours was not associated with decreased mortality compared to delayed clearance. The reasons for the lack of difference in mortality are speculative but may be related to the fact that patients who underwent early focus clearance were more severely ill at the time of the decision and, therefore, the effect of early intervention was underestimated due to the higher mortality of this group. In turn, the more severe presentation may be indicative of a late diagnosis in some patients in whom the septic process was already advanced and for whom earlier intervention did not have the expected effect.

In the most recent SSC publication, there was a revision of the ideal time for source control. Until 2012,^(14)^ there was guidance that the focus should be cleared in less than 12 hours, if possible, based on a study that evaluated patients with necrotizing fasciitis.^(15)^ In the most recent publication from 2016,^(10)^ it was recommended that septic focus clearance be performed as soon as possible, indicating the persistence of doubts regarding an ideal time for focus clearance. Another recent guideline recommends that patients with septic shock undergo emergency focus clearance, although it can be postponed in cases of lesser severity (recommendation 2C; weak recommendation and low or very low quality evidence), further emphasizing that the optimal time for septic focus control has not been rigorously investigated.^(16)^

These guidelines are in agreement with observational studies and expert opinion, which suggest that early focus clearance after the diagnosis of sepsis would result in lower mortality rates.^(17-19)^ Azuhata et al., in a prospective study, concluded that the implementation of a protocol for early control in 154 patients with gastrointestinal perforation was associated with a reduction in mortality by 60 days (OR = 0.31 per hour of delay). In that study, all patients underwent surgery in the first 6 hours.^(19)^ In another study, delayed surgical intervention for patients with perforated peptic ulcer was associated with an increase in mortality of 2% for every hour.^(20)^ It is noteworthy that, in general, these studies were observational, had small sample sizes that compromised the power of the studies and had limitations associated with external validity. Moreover, most of them were conducted in developed countries, in which organized health systems allow earlier intervention. As in our study, these studies exclusively evaluated patients with abdominal septic focus.

In a multicenter observational Spanish study,^(21)^ 1,090 patients underwent focus control procedures, and earlier control of the infectious focus (< 12 hours) was not associated with lower mortality, similar to the results found in our study. The authors argued that the teams considered focus control more urgent for the most severe patients, although the multivariate analysis was not able to show this effect. Both the results of that study and its discussion apply to our results.

Our study has several limitations. The total number of included patients was smaller than the calculated sample size, which limits the power of the study to show statistically significant results. There was no evaluation of the population of patients with abdominal sepsis who did not undergo focus control. This population includes patients with foci that are not amenable to control, such as gastroenteritis, and may include patients who died before focus control, introducing a potential selection bias in the study sample. Only patients admitted to the ICU were studied, and patients with satisfactory progression after septic focus clearance may have been excluded. We also did not assess whether the focus clearance was considered adequate. Although the time to septic focus clearance is based on the time when the diagnosis of sepsis was determined, which is subject to inaccuracies during data collection, there is no reason to infer the existence of an imbalance between groups in the collection of these data.

A strength of this study to be highlighted is the selection of a population of patients with sepsis and septic shock with abdominal focus, in contrast with other studies that have evaluated patients with cutaneous or thoracic foci and those with infection who do not meet criteria for sepsis or septic shock.

Although we found no significant difference in mortality between early and delayed intervention, based on current evidence, we do not believe that there is any reason to delay focus control. However, this study corroborates other studies recommending that in cases in which better clinical stabilization of the patient or better surgical planning is needed, focus control can be briefly delayed without negatively impacting mortality.

## CONCLUSION

There was no difference in mortality in patients with sepsis or septic shock who underwent abdominal septic focus clearance less than 12 hours after diagnosis. Clinical trials should be conducted to determine at what time point or in which patients septic focus clearance may be beneficial.
